# Deficiencies and How They Will Be Made Good

**Published:** 1913-12-20

**Authors:** 


### NATIONAL INSURANCE ACT DEVELOPMENTS.

### Deficiencies and How They Will be Made Good.

It is a recognised principle in all sound business concerns that the policy on which the business is to be carried on in the future must largely depend upon the result of the periodical balancing of accounts. If a deficit be revealed, measures of retrenchment must be decided upon and immediately carried out, whilst if a profit be shown the shareholders correspondingly benefit, and extensions of the business may be instituted. This principle applies in an equal degree to the affairs of approved societies. The periodical balancing of accounts is here represented by the successive valuations, and a deficit or a surplus is shown according as the estimated value of the liabilities exceeds that of the assets or *vice versa*.

We dealt last week with the probable effect of excessive sickness claims upon the result of the first valuation of the approved societies, and indicated that there were good reasons for supposing that such excess sickness was actually being experienced. Backed by other considerations, we were led to the general conclusion that this first valuation is likely to reveal an unsatisfactory position in many societies.

### Some Other Possible Causes.

It should also be pointed out that though excessive sickness was dealt with largely last week as a cause of deficiency, it is by no means the only possible cause; and considerable loss may result in other ways, some of which may be unexpected. Thus, for instance, maternity benefit may prove more costly than the estimate. It may be remarked in this connection that the data available for the calculation of the cost of this benefit was but slender, and in part had to be drawn from statistics relating to New Zealand. Again, the estimate was made for all insured persons, distinguishing necessarily between men and women; and while it may be applicable to the whole body of insured persons, it by no means follows that it will apply to individual societies, for reasons similar to those we instanced last week regarding the sickness experience of self-selected societies.

Moreover, disablement benefit has not yet become operative, and before the close of the valuation period it may become apparent that the excess claims for sickness benefit are but a foretaste of the adverse experience of the extended drain on the funds of the societies for reduced sick-pay. This may even be more serious than excess claims for sickness benefit, for few societies could afford to allow any number in excess of the expectation to remain permanently on the funds without feeling acutely the effects of the strain. Against all these possible sources of loss to approved societies, there is one source of profit which, it might be thought, would in some measure outweigh them. We refer to the possibility of earning a higher rate of interest on the funds than the actuaries assumed would be earned. We purpose dealing more fully with this question in a later article, and it will be sufficient for our present purpose to say that, for reasons we shall there give, it is improbable that much will be forthcoming from this source at present.

### The Remedy.

It is, therefore, of more than passing interest to consider the remedial measures that will have to be adopted to meet the situation, should any substantial number of societies be found to be in the unfortunate position that has been indicated. The procedure both for valuations and the distribution of the surpluses or the liquidation of the deficiencies that may be disclosed is laid down in the Act, but before considering this in detail it will be better to look at the general principles. Suppose, then, that a society has actually been shown to have a deficiency. What are the steps that should be taken? Undoubtedly the first thing to do will be to follow the example of any ordinary business enterprise by endeavouring to ascertain how the deficiency has arisen and where the leakage has occurred. This will be beneficial in many ways. For one thing a careful study of the result of this investigation will go a long way in assisting the society to come to a decision as to the particular remedial measures to be adopted.

Equity demands that two fundamental principles should be observed in dealing with a realised deficiency—namely, (1) that a recurrence of the deficiency be guarded against; (2) that the burden of making good the accrued deficiency be borne, as far as possible, by that body of members among whom it has arisen.

The absolute necessity of inquiring into the causes that produced the deficiency will be clearly seen if the first of the above principles be considered. Beyond observing that it is as important, if not more important, to prevent future loss as to remedy past deficiency, it is not proposed to dwell any further here on principle (1).

The equity of the second of the above principles will be obvious to even the most casual observer, but its strict application in practice is a good deal more difficult than might be thought, and at best it can only be hoped to mete out rough justice to the members generally.

### Separation of Old and New Members.

One main division of the members may, however, in any case be undertaken. Members who join after the deficiency has arisen should clearly not be called upon to share the burden of it, except in perhaps a very small degree, and it is clear that separate treatment must be given to the contracts of the new and the old members. The main consideration with respect to new members would be that the contributions paid by them should be adequate to pay for the benefits obtained and a due proportion of the expenses. Societies will, of course, be automatically prevented from going much further than this, since any attempt to penalise new members would immediately result in keeping new entrants away altogether, and thus the attempt would bring about its own defeat. An understanding of the measures that best carry out principle (2) with respect to old members may perhaps be obtained in the most satisfactory way by considering the requirements of the Act of 1911 regarding societies in deficiency.

### Way of Making up Deficiencies.

After dealing with the course to be adopted when the society is a branch and not an independent society, and for the grouping of small societies, the Act goes on to direct that a scheme for making good the deficiency shall be submitted to the Commissioners and that such scheme shall provide for making good the deficiency within a period of three years from the date at which the valuation was made in any one or more of the following ways:

(1) By a compulsory levy, by way of increase of the weekly rate of contribution upon members of the society being insured persons; (2) by reducing the rate of sickness benefit either for the whole period during which sickness benefit is payable or for any part thereof; (3) by deferring the day as from which sickness benefit becomes payable; (4) by reducing the period during which sickness benefit is payable; (5) by increasing the period which is required to elapse between two periods of disease or disablement to prevent the one being treated as a continuation of the other.

If the deficiency be small and be due to a cause, such as inefficient supervision of claims or depreciation of investments, which cannot be laid to the charge of any particular section of the members, the most equitable way of dealing with the matter so far as the accrued deficiency is concerned would be to wipe out the loss by a single levy or succession of levies, every member contributing the same amount. In the alternative some such measure as (3), (4), or (5) might be adopted, which roughly treats all members alike.

### Position of Younger Members.

If the deficiency can be traced to any cause for which the younger members are mainly responsible a permanent increase in contribution would be more suitable, as if the same increase were charged to all members the younger members, who have to pay contributions for a longer period, would be penalised to a greater extent. It might be thought that a general reduction of benefit, such as suggested in (2), would meet the case equally well, but it must be remembered that this would press more hardly on the old members, among whom a far greater proportion are in receipt of sickness or disablement benefit. It can be seen that the younger members are as a whole likely to survive the present period of difficulty in which their society is placed, and thus possess a chance, not shared by the older members, of living to a period of prosperity of the society which will outweigh any present loss. It would be out of place to consider more in detail at the present moment schemes to be adopted in particular circumstances, as this can well be left until the results of the first valuation are made known.

### Prompt Action to be Taken.

What we wish to point out here is that if from any cause a deficiency is revealed when the first valuation is made immediate steps will have to be taken to place the society in a solvent position, and, moreover, any scheme devised must allow for making good the deficiency *within three years.* A society cannot hope to evade its obligations in this direction by merely letting things slide, as it is provided by the Act that if a scheme for remedying the deficiency be not submitted to the Commissioners within six months of its being revealed, or if at any time thereafter it appears to the Commissioners that the society to which the scheme relates is not enforcing the provisions of the scheme, the Commissioners may take over the administration of the affairs of the society and must, as soon as possible, enforce measures to make good the deficiency.

Approved societies are thus under more stringent control than that exercised over the old friendly societies and will be unable to neglect a deficiency for so many years and to such an extent as was done by so many friendly societies with such disastrous results. This stringent supervision is undoubtedly to the great advantage of the societies as a whole, as it will tend to keep them in a sound and healthy condition, but it is not always easy to convince a person that a present hardship is for his ultimate good, and it may therefore be anticipated that there will be a good deal of grumbling among members if the first valuation reveals an unsatisfactory position.

### Individual not to Escape.

In addition to the safeguards that have been set up to ensure that the society shall not shirk its responsibility it has been made equally sure that the individual member shall not escape his share of the burden by merely transferring his membership to another society. It is enacted that any member who having been a member at the date as at which the valuation disclosing the deficiency was made is transferred to another society before the deficiency is made good shall be liable to any levy, reduction of benefit, etc., in precisely the same manner as if he had remained a member of the old society. It may be wondered why we have dealt at such length with deficiencies and their results, and we would point out in reply that to be forewarned is to be fore-armed, and that it is infinitely better to prevent the occurrence of a deficiency than to remedy it when it occurs. Insured persons should therefore follow intelligently the affairs of their respective societies and, having a knowledge of possible sources of loss, endeavour to see that such loss is as far as possible prevented.

